# Evaluation of lymph node findings in patients with and without odontogenic infection: A clinical and ultrasonographic study

**DOI:** 10.4317/medoral.26170

**Published:** 2023-10-12

**Authors:** Nuray Bagci, Ilkay Peker, Tuba Gündüz

**Affiliations:** 1 Department of Dentomaxillofacial Radiology, Faculty of Dentistry, Gazi University, Ankara, Turkey; 2 Department of Measurement and Evaluation in Education, Faculty of Education, Muğla Sıtkı Koçman University, Muğla, Turkey

## Abstract

**Background:**

The present study aimed to evaluate the ultrasonographic findings of submandibular and submental lymph nodes in patients with and without odontogenic infection.

**Material and Methods:**

Systemically healthy patients aged 18-30 years old with or without odontogenic infections were included in this study. Clinical examinations were performed on all patients; those with any odontogenic infection were placed in the study group, and those without were placed in the control group. Ultrasonographic examinations of bilateral submental and submandibular lymph nodes were performed for both groups. The data were statistically analyzed using Pearson’s Chi-square test and Student’s t-test.

**Results:**

A total of 150 patients voluntarily participated (female: *n*=86 (57%), male: *n*=64 (43%)), 75 in the study group and 75 in the control group. During the ultrasonographic examination, patients in the study group had more than one lymph node the same patient was mostly detected, in the study group (right submandibular: *n*=42, 56%, and left submandibular: *n*=43, 57.3%). The long-axis diameter of the submandibular lymph nodes was 9.305.30 mm and 5.505.20 mm in the study and control groups, respectively.

**Conclusions:**

Ultrasonography revealed that the presence, number, and long-axis diameter of the submandibular lymph nodes in the patients with and without odontogenic infection were statistically different.

** Key words:**Odontogenic infection, lymph node, ultrasonography.

## Introduction

The lymphatic system is one of the body’s most important immune system. This system protects the body against various stimuli, such as microbial agents, chemicals, tissue injuries, infections, immune complexes, and neoplasms (1). Lymph nodes constitute a large part of the lymphatic system and are distributed throughout the body (2). The nodes are oval-shaped structures comprising a medulla and a cortex surrounded by a fibrous capsule; the hilum in the medulla comprises lymphoid sinuses, arteries, veins, and fatty tissue. These nodes provide lymph drainage to the nearest tissue (1).

The cervical lymph nodes are generally not palpable during clinical examination in healthy individuals. Lymphadenopathy (LAP) is defined as clinically detecTable swelling of the lymph nodes. LAP can occur in various conditions, such as infection, immunological disorders, tuberculosis, and malignancy. Specifically, the submandibular and submental cervical lymph nodes become reactive and increase in number and size due to oral, throat, and odontogenic infections (2). LAP can be examined by imaging methods in addition to clinical detection (3).

Ultrasonography, Computed Tomography (CT), and Magnetic Resonance Imaging (MRI) are used to examine the lymph nodes in both healthy and diseased states. Each imaging method has different advantages and benefits. Ultrasonography is more advantageous than CT for imaging small the lymph nodes. The lymph node diagnostics by MRI is challenging if calcification is present (3). Ultrasonography is more sensitivity for accurate pretreatment staging and the detection of the lymph node involvement than Positron Emission Tomography (PET), MRI, and CT procedures in patients with oral malignancies (3). Ultrasonography is non-invasive, radiation-free, easy to apply, repeaTable, can obtain real-time images, and has high sensitivity and specificity in the lymph node imaging. The lymph node size, structure, borders, hilum, and vascularization can be examined by ultrasonography, as can calcification or necrosis (1).

Many ultrasonography, CT, and MRI studies reveal changes in the lymph nodes, such as infections, tuberculosis, benign lesions, and malignancy (1,3-9). Differences in the lymph nodes of patients with odontogenic infections versus those without have not been examined by ultrasonography before. The present study aimed to evaluate the ultrasonographic findings of the submandibular and submental lymph nodes in the patients with and without odontogenic infection.

## Material and Methods

This study was approved by the Gazi University Faculty of Dentistry Ethics Committee (Document Date-Number 30.01.2020-GÜDHKAEK.2020.03/7) and complied with the Declaration of Helsinki. Written informed consent was obtained from all volunteers.

Patients who visited the Oral Diagnosis Clinic within the Faculty of Dentistry at University for odontogenic problems between October 2021 and April 2022 were included in this study. The inclusion criteria for selecting study patients were age 18-30 years, systemically healthy, and with/without odontogenic infections. Those with infections other than odontogenic infection or diseases that can cause LAP (infection, immunological, malignancy, lipid storage disorder, endocrine, and other diseases) were excluded (2).

The patients first underwent clinical examinations, during which demographic characteristics (age and sex) and systemic anamnesis were recorded, and extraoral, intraoral, and radiographical examinations were performed. Panoramic (Sirona-Orthophos XG; Sirona; 60-90 kVp; 8 mA; 14 seconds) or periapical (CCX radiography unit, Tropy, Instrumentarium, Tuusula, Finlandiya; 70 kVp; 8 mA; 0.3 seconds) radiographs were taken per the indication. The examination results were used to divide the patients into the study and control groups. The patients with odontogenic infections constituted the study group and those without constituted the control group. Dental abscess, acute or chronic apical periodontitis, periodontitis, pericoronitis, and residual dental radix were considered odontogenic infections (1).

**Figure 1:**
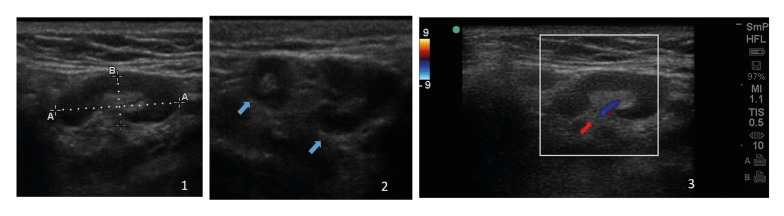
Ultrasonographic images (1: B-mode ultrasonographic appearance and size measurements of a submandibular lymph node, 2: Two different lymph nodes in a single image, blue arrows, and 3: Doppler ultrasound scanning of a submandibular lymph node with hilar vascularity, red arrow).

Ultrasonographic examinations were then applied for all patients using a SonoSite M-Turbo ultrasound device (FUJIFILM SonoSite Inc., 21919 30th Drive SE Bothell, WA 98021 USA) and a 6-13 MHz linear array transducer. Ultrasonographic images were obtained in gray-scale by B-mode and Doppler ultrasound. The presence, number, localization, diameter, boundary, shape, echogenicity, and hilum of the lymph nodes was assessed. The Doppler ultrasound was used to assess the intra-nodal vascularity pattern (Fig. 1). During the examination, the bilateral submental and submandibular lymph nodes were evaluated according to Hajek’s Classification (10). The examinations were performed on the patients in the supine position.

**Figure 2:**
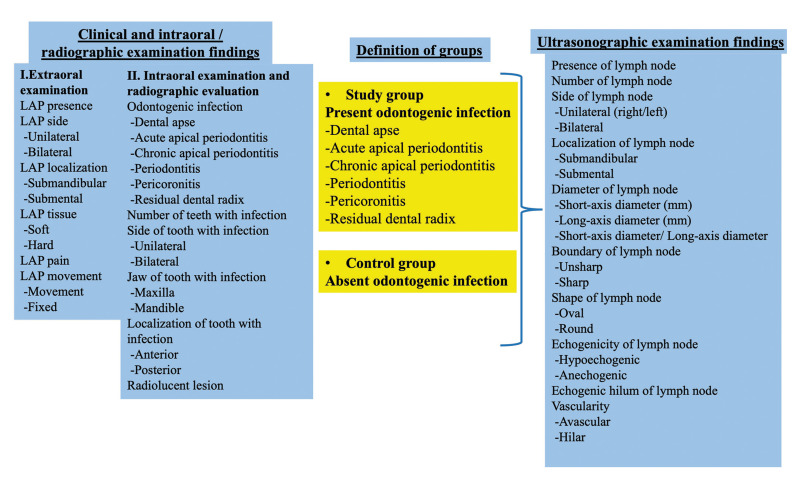
Variables evaluated in the study and the group definitions.

The patient’s neck was hyperextended, and the head was turned to the opposite of the examination side (9). Thus, the examined side was tense for optimally visualizing the lymph nodes. An oral and maxillofacial radiologist with at least four years of experience performed the clinical and ultrasonographic examinations. The clinical and ultrasonographic examination findings were recorded and are presented in this study (Fig. 2).

- Statistical analyses

Power analysis was performed in the G-Power 3.1.9.4 program to determine the minimum sample size (11). The required sample size was determined to be 70 per group (error probability; 0.05, effect size; 0.5, power; 0.90).

 The statistical analyses were conducted with SPSS® software (SPSS v. 20.0 for Windows, SPSS Inc., Chicago, IL). Descriptive analyses were performed, and the frequencies were calculated. The presence of LAP was analyzed using the Pearson’s chi-square test to assess difference between groups. The LAP number and long-axis diameter was analyzed using an independent Student’s t-test to assess in difference between groups. *P*<0.05 was considered to represent significant.

## Results

A total of 150 patients were included (study group: 75 patients; control group: 75 patients), aged 18-30 years (mean standard deviation: 24.18 3.16) (Table 1).

Table 2 shows the clinical examination findings of the study group. Submandibular LAP was detected in four patients during the extraoral examination. Chronic apical periodontitis was the most common odontogenic infection observed by the clinical examination.

Table 3 shows the ultrasonographic examination findings per group. The detection of more than one lymph node in the same patient was common in the study group (*n*=42, 56%, and *n*=43, 57.3%; right submandibular and left submandibular, respectively). Only one lymph node was detected per patient in the control group. In addition, the left submental lymph node was not detected in either group.

The ultrasonographic examination revealed that the presence and number of the right and left submandibular lymph nodes were statistically higher in the study group than in the control group. In addition, the mean of the long-axis diameters of the right submandibular lymph node was higher in the study group. This mean differed significantly between the two groups (Table 4).

**Table 1:**
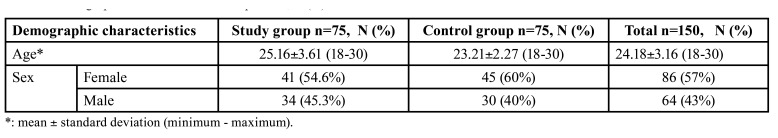
Demographic characteristics of the patients, N (%).

**Table 2:**
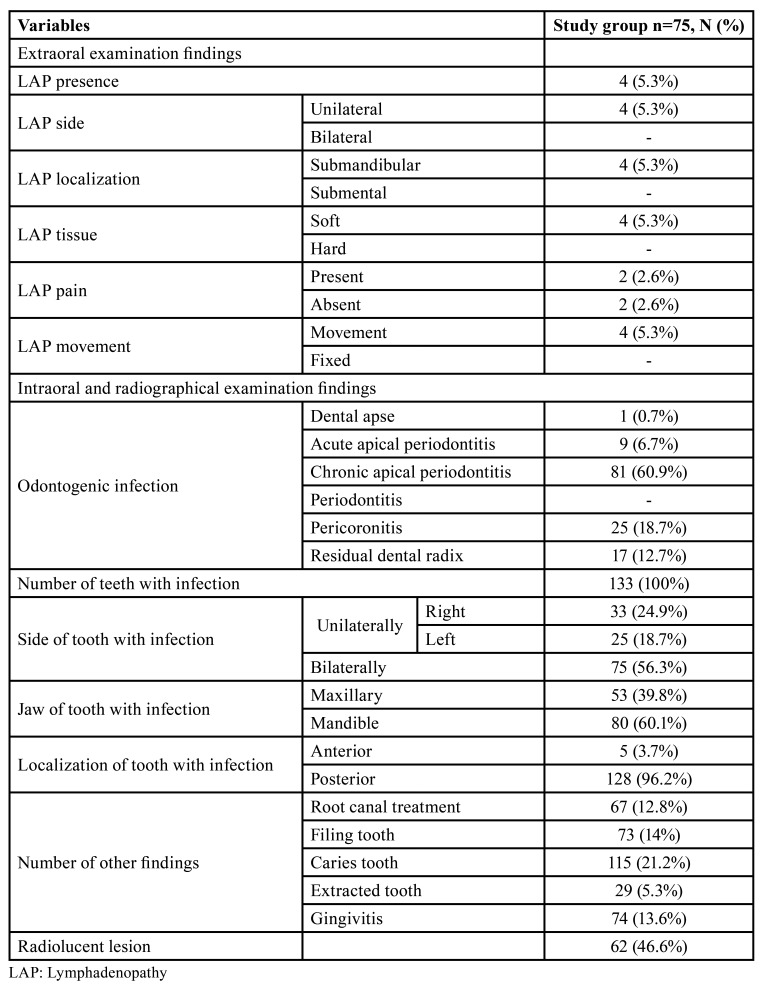
Distribution of the clinical examination findings in the study group, N (%).

**Table 3:**
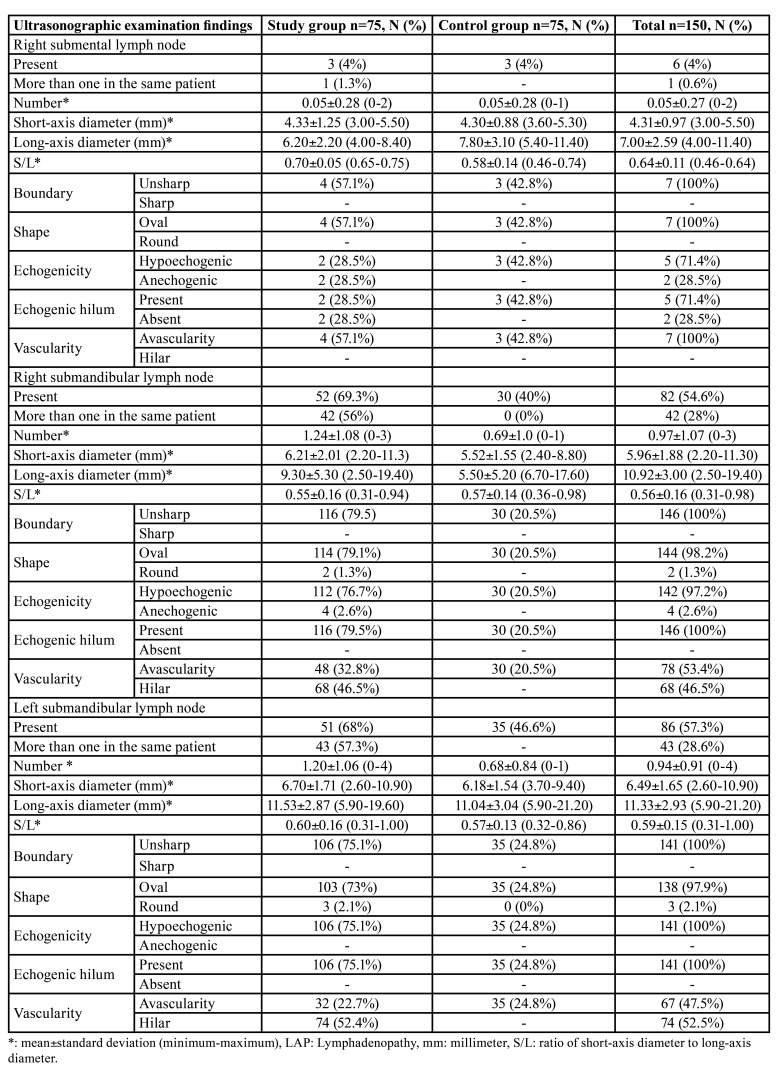
Distribution of the ultrasonographic examination findings according to the groups, N (%).

**Table 4:**
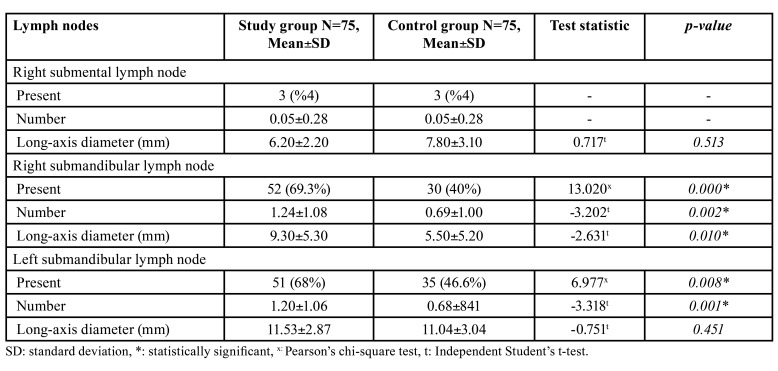
Statistical analysis of the ultrasonographic examination findings according to the groups.

## Discussion

In the present study, the clinical and ultrasonographic examinations of the submental and submandibular lymph nodes were conducted on the patients with and without odontogenic infection. The presented ultrasonographic examinations demonstrate that the presence, number, and long-axis diameter of the lymph nodes differed significantly between the patients with and without odontogenic infection.

Different imaging methods have been used to examine the lymph nodes (3). Jun Seok *et al*. reported that MRI is insufficient for detecting the small lymph nodes (3 mm) in patients with metastasis (12). Ishii *et al*. reported that CT is insufficient to scan the lymph nodes smaller than 5 mm (13). Okumuş *et al*. applied ultrasonography and found that the smallest-axis diameters of the submandibular and submental lymph nodes are 1.8 and 2.3 mm (14). In the present study, the smallest-axis diameters of the submandibular and submental lymph nodes were 2.2 and 3 mm. Thus, ultrasonography can be useful for detecting the small lymph nodes.

In healthy individuals, the size of the lymph nodes in the cervical region can vary from 3 mm to 3 cm (15). Ying *et al*. found that the mean long-axis diameter of the submandibular lymph node was 7.6 2.2 mm in healthy males and 6.7 1.6 mm in healthy females (16). Okumuş *et al*. found that in healthy individuals, the mean long-axis diameters of the right and left submandibular lymph nodes were 12.84 4.4 mm and 11.94 ± 1.95 mm, respectively (14). In the present study, the mean long-axis diameters of the right and left submandibular lymph nodes were 5.50 5.20 and 11.04 3.04 mm in the control group and 9.30 5.30 and 11.53 2.87 mm in the study group. The mean long-axis diameter of the submandibular lymph node was in line with previous studies.

The lymph node diameter increases in certain diseases (2). Rue *et al*. reported that the mean longest size of the cervical lymph nodes was significantly larger in patients with malignant diseases than in those with benign conditions (17). Lakshmi *et al*. found that the mean maximum transverse diameter of the cervical lymph nodes was significantly higher in patients with odontogenic infections and malignant symptoms than in healthy individuals (9). The mean long-axis diameter of the right submandibular lymph node was statistically higher in the study group than in the control group in the present study. Consistent with previous studies, the size of the affected lymph nodes in infected patients increased.

The ratio of the large to the small-axis diameter can provide information regarding normal and pathologic lymph nodes (18). This ratio has been used to differentiate the normal or reactive lymph nodes from a malignant lymph nodes. The ratio of the normal cervical lymph nodes is under 0.5, the ratio of a tuberculous lymph nodes is greater than 0.5, and the ratio of the lymph nodes with odontogenic infection is 0.47 (1,14). A rate 0.67 and above confirms a metastatic lymph node with a probability of 70.44% (5). In the present study, the mean ratio of the right and left submandibular lymph nodes with odontogenic infection were 0.55 and 0.60, respectively. The mean ratio of the submandibular lymph nodes without odontogenic infection was 0.57. Small differences in these ratios may results from sample and practitioner differences between studies and the lymph node anatomy.

The lymph node shape can be oval or round, and the shape can provide clues regarding pathological conditions (18). It has been reported that the lymph node shape is generally oval in healthy individuals, and the submandibular and parotid lymph nodes are round (19). Previous studies have reported that the normal and reactive lymph nodes are oval, while the malignant and tuberculous lymph nodes are round (1). A different study found that the cervical lymph nodes are oval in all oral squamous cell carcinoma cases (5). In the present study, the shape of the submandibular and submental lymph nodes was mostly oval in both healthy and odontogenic infected individuals. Thus, the ultrasonographic shape of the lymph nodes should not be the only pathological criterion; the patient’s history and the clinical findings should be evaluated.

On ultrasonographic examination, the normal and reactive lymph nodes have unsharp borders due to the associated oedema and inflammation in the surrounding soft tissues (18). The nodes have echogenic hilum with a hyperechoic linear structure. In addition, because the nodes are nourished by the hilum, hilar vascularity is observed (19). In the present study, all submandibular lymph nodes were found to have unsharp borders and echogenic hilum both in the control and study groups. In addition, the vascularity of the bilateral submandibular lymph nodes was mostly hilar in the study group and avascular in the entire control group. These findings were consistent with previous studies (1,7,8,20).

This study had some limitations. Although odontogenic infections first drain to the submandibular lymphatics, they may later drain into the deep cervical lymph nodes. Therefore, in long-term untreated odontogenic infections, examining only the submandibular lymph node is insufficient, and imaging of the deep cervical lymph nodes can also be performed.

## Conclusions

In the present study, the ultrasonographic findings of the submandibular lymph nodes varied between the patients with and without odontogenic infection. On the ultrasonographic examination, significant differences were found between the study and control groups in the presence, number, and long-axis diameter of the submandibular lymph nodes.

Ultrasonography can examine the submandibular and submental lymph nodes in the patients with odontogenic infections that cannot be detected by the clinical examination. Notably, it is important to remember that the submandibular and submental lymph nodes detected incidentally during neck ultrasonography performed for various reasons may be associated with odontogenic infections. In addition, objective discriminations of the inflammatory and normal lymph nodes can be conducted using ultrasonography images.
